# Supplementary figures and images for: Modeling the effects of water temperature on the population dynamics of Galba viatrix and infection by Fasciola hepatica: a two-year survey in Andean Patagonia, Argentina
Source: PeerJ. 2024 Dec 20;12:e18648. doi: 10.7717/peerj.18648 (PMC11665429; doi:10.7717/peerj.18648)

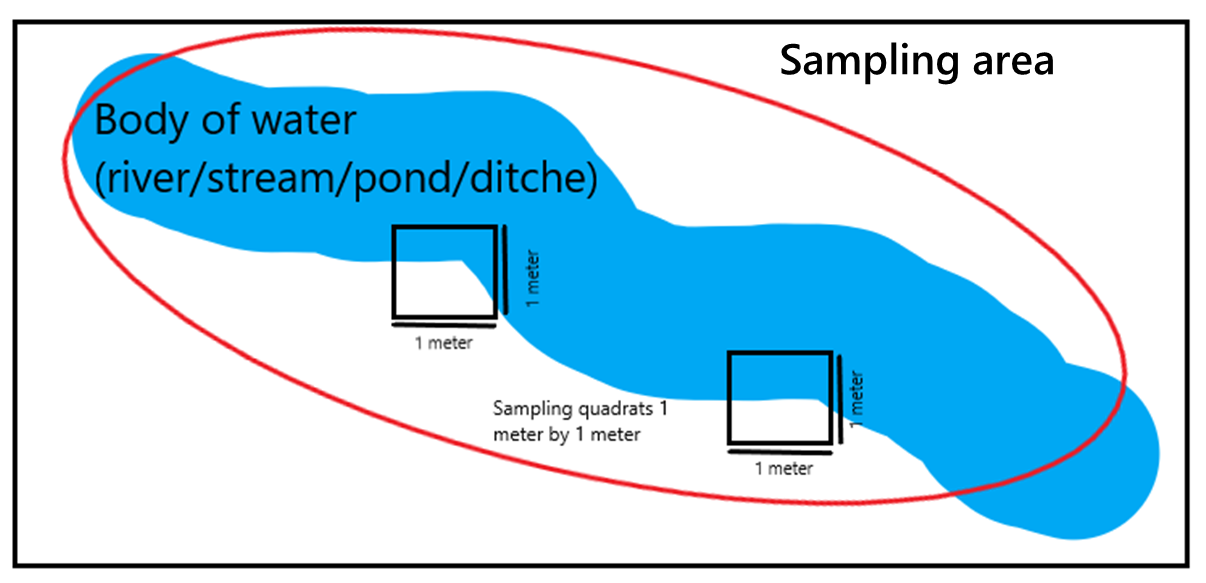

Supplement: Supplemental Information 1 — General outline of the method of sampling, at each of the four selected sites, 2-3 quadrants were delimited for snail sampling and the geographical coordinates recorded. [file peerj-12-18648-s001.png]

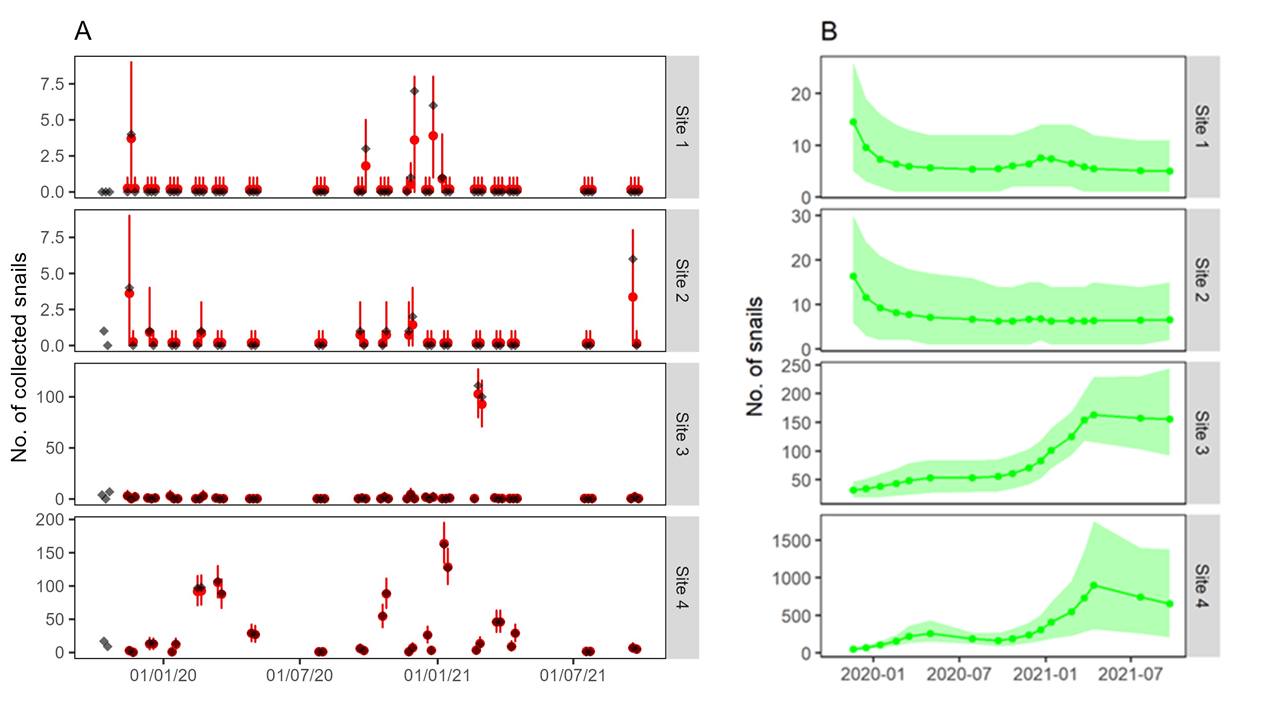

Supplement: Supplemental Information 2 — (A) Observed and predicted number of collected snails. The black points represent the number of collected snails. The red points and error bars indicate the model prediction of the mean observed number of snails and their 90 % interquantile range. Each point corresponds to a quadrant within each survey date. (B) Estimated “real” population size of snails. The green line corresponds to the mean predicted “true” number of snails in each population. The shaded areas indicate the 90 % interquantile range of the corresponding posterior distributions of the fitted model. Each row of plots corresponds to a sampling site. [file peerj-12-18648-s002.png]

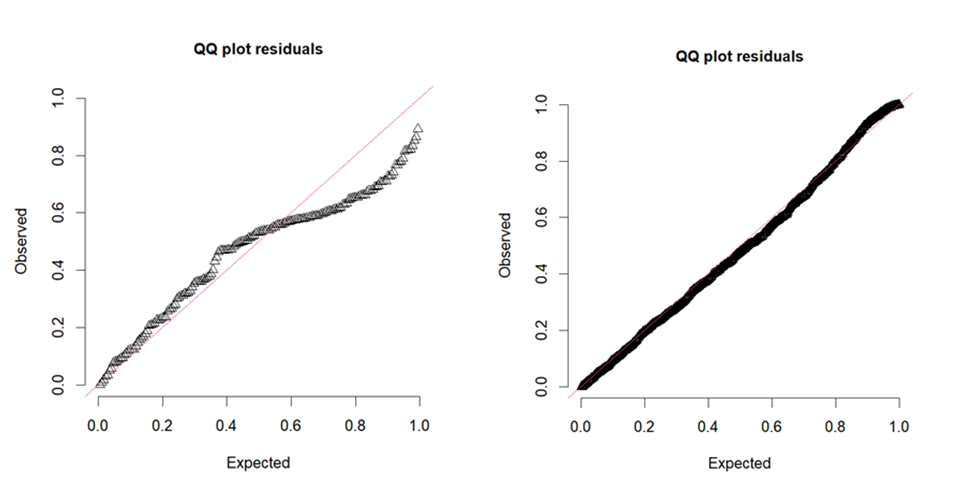

Supplement: Supplemental Information 3 — These Q-Q plots assess how much the values predicted by the model (in our case, the posterior predicted distribution) differ from the observed data. (A) Q-Q plot for population dynamics model. (B) Q-Q plot for infection model. [file peerj-12-18648-s003.png]

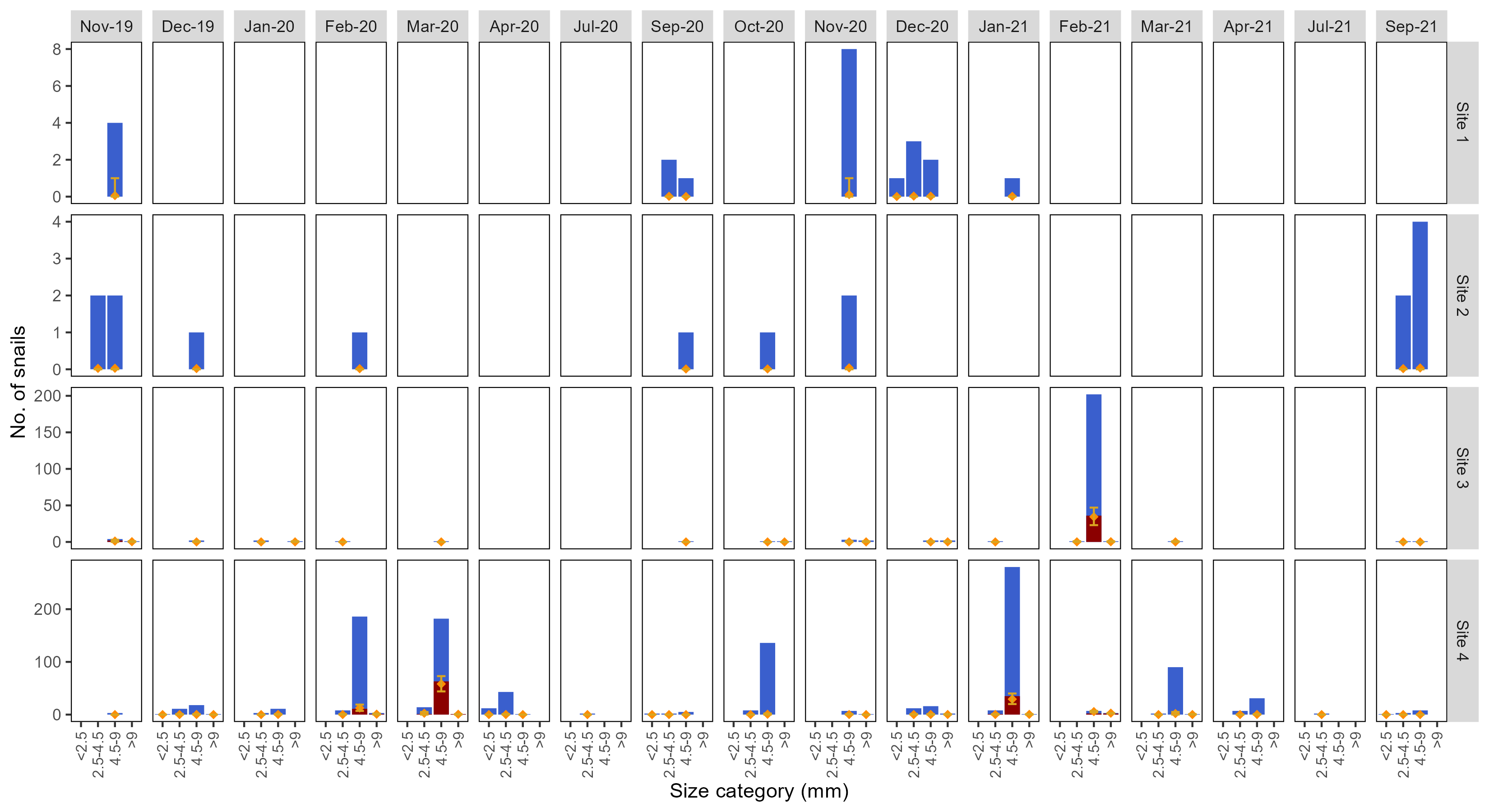

Supplement: Supplemental Information 4 — Bars in red indicate the number of infected snails and in blue, uninfected. Orange points and error bars indicate the hierarchical model predictions and their 90 % interquantile range. [file peerj-12-18648-s004.png]
