# Supplementary material for: Modeling the effects of water temperature on the population dynamics of Galba viatrix and infection by Fasciola hepatica: a two-year survey in Andean Patagonia, Argentina
Source: PeerJ. 2024 Dec 20;12:e18648. doi: 10.7717/peerj.18648 (PMC11665429; doi:10.7717/peerj.18648)
Supplement: Supplemental Information 5 [file peerj-12-18648-s005.docx]

Equations S1. Full specification of models and justification of their priors

**Population dynamics model:**

$$y_{t,j,k}\sim BetaBinomial\left( p_{t,j,k},Y_{t,j},\phi\right)$$

$$\mathrm{logit}\left( p_{t,j,k} \right)=g+hT_{t,j,k}^{w}$$

$$Y_{t,j}\sim Poisson\left( \lambda_{t,j} \right)$$

$$\lambda_{t,j}=\beta_{t,j}Y_{t-1,j}+\gamma$$

$$\beta_{t,j}=\frac{\kappa_{j}}{\left( 1+e^{\alpha T_{t,j}^{m}+\delta} \right)}$$

$$\kappa_{j}\sim Normal\left( \mu_{\kappa},\sigma_{\kappa}^{2} \right)$$

$$\phi\sim Normal\left( 100, 100 \right)T\left( 0, \right)$$

$$g\sim Normal(-10, 4)$$

$$h\sim Normal(0.5, 0.01)$$

$$\gamma\sim Exponential(0.5)$$

$$\alpha\sim Normal(0.3, 0.01)$$

$$\delta\sim Normal(0, 1)$$

$$\mu_{\kappa}\sim\mathrm{Exponential}(1)$$

$$\sigma_{\kappa}\sim\mathrm{Exponential}(3)$$

$\phi$: The higher the value of this dispersion parameter, the lower the overdispersion with respect to a binomial distribution. We opted for a prior with high mean to ensure that the model considered overdispersion only if data suggested it strongly enough.

$g$: This prior was rather informative since the model could have more difficulty to successfully converge if the estimation of the observation probability (and, thus, of the latent variable) was too nonrestrictive. Based on previous field observations and reports (Claxton et al., 1999; Kleiman et al., 2007; Bargues et al., 2021), we considered that temperatures below 10 °C would strongly hinder finding snails.

$h$: Based on previous field observations of ours and reports (Ollerenshaw, 1959; Bargues et al., 2021), we considered that temperature would have a positive effect on detection probability, but we allowed enough variability.

$\gamma$: A non-negative prior that allowed enough variability.

$\alpha$: We expected the effect of temperature to be positive on the population growth rate (Ollerenshaw, 1959; Bargues et al., 2021). Thus, we chose a prior with a mean >0, allowing considerable variation.

$\delta$: We chose this weakly informative prior to allow the logistic function to be flexible enough to fit the data.

$\mu_{\kappa}$: A non-negative prior that allowed enough variability around unity value (i.e., invariant population size).

$\sigma_{\kappa}$: A non-negative prior that allowed enough variability.
