# Supplementary material for: Modeling the effects of water temperature on the population dynamics of Galba viatrix and infection by Fasciola hepatica: a two-year survey in Andean Patagonia, Argentina
Source: PeerJ. 2024 Dec 20;12:e18648. doi: 10.7717/peerj.18648 (PMC11665429; doi:10.7717/peerj.18648)
Supplement: Supplemental Information 6 [file peerj-12-18648-s006.docx]

**Supplementary Material**

Equations S2. Infection dynamics model.

$$z_{i,j}\sim Bernoulli\left( q_{i,j} \right)$$

$$\mathrm{logit}\left( q_{i,j} \right)=a_{j}+b_{s}s_{i,j}+b_{f}f_{i,j}+b_{T}T_{i,j}^{m}+b_{\mathrm{sf}}s_{i,j}f_{i,j}+b_{\mathrm{sT}}s_{i,j}T_{i,j}^{m}+b_{\mathrm{fT}}f_{i,j}T_{i,j}^{m}+b_{\mathrm{sfT}}s_{i,j}f_{i,j}T_{i,j}^{m}$$

$$a_{j}\sim\mathrm{Normal}\left( -3,1 \right)$$

$$b_{s}, b_{f},b_{T},b_{\mathrm{sf},}b_{\mathrm{sT}},b_{\mathrm{fT}},b_{\mathrm{sfT}}\sim\mathrm{Normal}(0, 5)$$

$a_{j}$: We expected the effects of the variables considered would imply midpoint of the logistic curve at a value >0 since all the considered variables were usually >0.

$b_{s}, b_{f},b_{T},b_{\mathrm{sf},}b_{\mathrm{sT}},b_{\mathrm{fT}},b_{\mathrm{sfT}}$: Although we expected all the considered variables to have positive effects on infection probability, we had no clear previous information on the magnitude of such effects. Thus, we chose this very non-restrictive normal prior.
